# Enhancement of acarbose production by genetic engineering and fed-batch fermentation strategy in *Actinoplanes* sp. SIPI12-34

**DOI:** 10.1186/s12934-022-01969-0

**Published:** 2022-11-23

**Authors:** Zhenxin Li, Songbai Yang, Zhengyu Zhang, Yuanjie Wu, Jiawei Tang, Luoju Wang, Shaoxin Chen

**Affiliations:** 1 State Key Laboratory of New Drug and Pharmaceutical Process, China State Institute of Pharmaceutical Industry, Shanghai Institute of Pharmaceutical Industry, Shanghai, 201203 China; 2 Department of Biological Medicines & Shanghai Engineering Research Center of Immunotherapeutics, School of Pharmacy, Fudan University, Shanghai, 201203 China; 3 Shandong Qilu King-Pharmaceutical Co., Ltd, No. 21 Qinglong Road, Pingyin County, Jinan, China

**Keywords:** Acarbose, *Actinoplanes* sp*.*, Transcriptome analysis, Genetic engineering, Multiple strategies, Fed-batch fermentation

## Abstract

**Background:**

Acarbose, as an alpha-glucosidase inhibitor, is widely used clinically to treat type II diabetes. In its industrial production, *Actinoplanes* sp. SE50/110 is used as the production strain. Lack of research on its regulatory mechanisms and unexplored gene targets are major obstacles to rational strain design. Here, transcriptome sequencing was applied to uncover more gene targets and rational genetic engineering was performed to increase acarbose production.

**Results:**

In this study, with the help of transcriptome information, a TetR family regulator (*TetR1*) was identified and confirmed to have a positive effect on the synthesis of acarbose by promoting the expression of *acbB* and *acbD*. Some genes with low expression levels in the acarbose biosynthesis gene cluster were overexpressed and this resulted in a significant increase in acarbose yield. In addition, the regulation of metabolic pathways was performed to retain more glucose-1-phosphate for acarbose synthesis by weakening the glycogen synthesis pathway and strengthening the glycogen degradation pathway. Eventually, with a combination of multiple strategies and fed-batch fermentation, the yield of acarbose in the engineered strain increased 58% compared to the parent strain, reaching 8.04 g/L, which is the highest fermentation titer reported.

**Conclusions:**

In our research, acarbose production had been effectively and steadily improved through genetic engineering based on transcriptome analysis and fed-batch culture strategy.

**Graphical Abstract:**

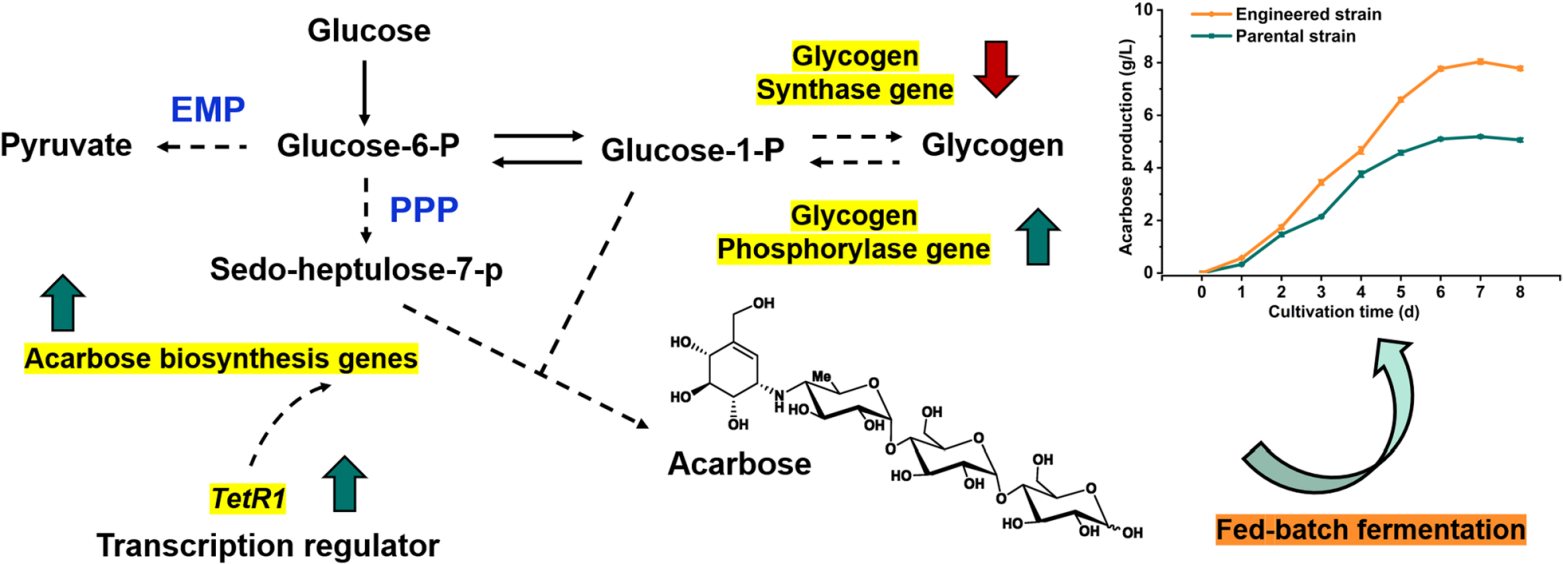

**Supplementary Information:**

The online version contains supplementary material available at 10.1186/s12934-022-01969-0.

## Background

Acarbose has been used widely to treat type II diabetes mellitus since the 1990s [1]. Because of its strong affinity for amylase and alpha-glucosidase in the intestine, acarbose can be used as a competitive substrate to decelerate the hydrolysis of starch and polysaccharides, delaying the generation of glucose to control the level of blood sugar after meals [2]. As the prevalence of type II diabetes is rapidly rising worldwide, an increasing demand for acarbose has to be anticipated [3].

At present, the large-scale industrial production of acarbose mainly relies on microbial fermentation. *Actinoplanes* sp., as the production strain, has been continuously optimized by conventional mutagenesis and screening procedures [4]. The complete genome sequence of *Actinoplanes* sp. SE50/110 was reported, with a characteristic feature of a high G + C content of 71.31% [5]. Relying on rapid advances in DNA sequencing technology, a large amount of information about the genome annotation [6, 7] and acarbose biosynthetic gene cluster (BGC) were revealed, and the metabolism of acarbose received a more detailed description [8–13]. For the acarbose biosynthetic process [14], Zhao redefined the synthetic pathway through physiological and biochemical studies and identified two shunt products derived from the synthesis process [15]. Recently, Takeshi Tsunoda further verified the pathway without supporting evidence and completely elucidated the whole process of acarbose biosynthesis on the basis of previous studies [16]. To date, the metabolic process of acarbose has been basically revealed, but limited knowledge about its regulatory mechanism still hinders the development of genetic engineering.

Based on the study of genomic information, Lena Schaffert, Tetiana Gren, etc., developed some effective strategies [17–19] and accessible toolkits [20, 21] for genetically engineering of *Actinoplanes* sp. Afterward, the first transcription factor regulating acarbose BGC, *AcrC*, was identified by Timo Wolf, which was an important step in the exploration of the regulatory mechanism of acarbose synthesis [22]. Taking advantage of the establishment of an efficient genetic manipulation system, a cloned acarbose BGC was introduced into *Actinoplanes* sp. SE50/110, resulting in an improvement in acarbose production [23]. During the synthesis of acarbose, the production of the C component not only affected the quality but was also difficult to be removed during its purification. Deletion of *treY* eliminated the C component [23, 24]. With the help of comparative functional genomics, Xie discovered and validated two potential gene targets (*ACWT_4325* and *ACWT_7629*), which encode alcohol dehydrogenase and elongation factor G, respectively. Deletion and complementation demonstrated their negativity for acarbose synthesis, and simultaneous deletion of the two genes resulted in an improvement of yield to 4.21 g/L [25]. Zhao further reduced the flux to shunt products and enhanced the supply of the amino-deoxyhexose moiety by genetic engineering, and acarbose production increased to 7.4 g/L [15]. Due to the uncertainty and inefficiency of mutagenesis, genetic engineering has an irreplaceable position and needs further exploration.

Omics approaches have been widely applied to explore the mechanisms of high-yield producers and target genes for genetic manipulation. To discover potential genes in *Actinoplanes* sp. SIPI12-34 (SIPI12-34), transcriptome sequencing was conducted at different fermentation times. Through the guidance of the transcriptome, we found a TetR family transcriptional regulator (TFR) with a positive effect on acarbose synthesis and some low expression level genes in the acarbose BGC. Combined with regulation of the metabolic pathways, the genes encoding glycogen synthase and glycogen phosphorylase were taken as targets for genetic manipulation to supply more precursors for the synthesis of acarbose. Finally, the production capacity of the engineered strain was further improved by fed-batch strategy.

## Results

### Regulation of transcriptional level to increase acarbose production

#### *TetR1* acts as a positive regulator of acarbose synthesis

**Fig. 1:**
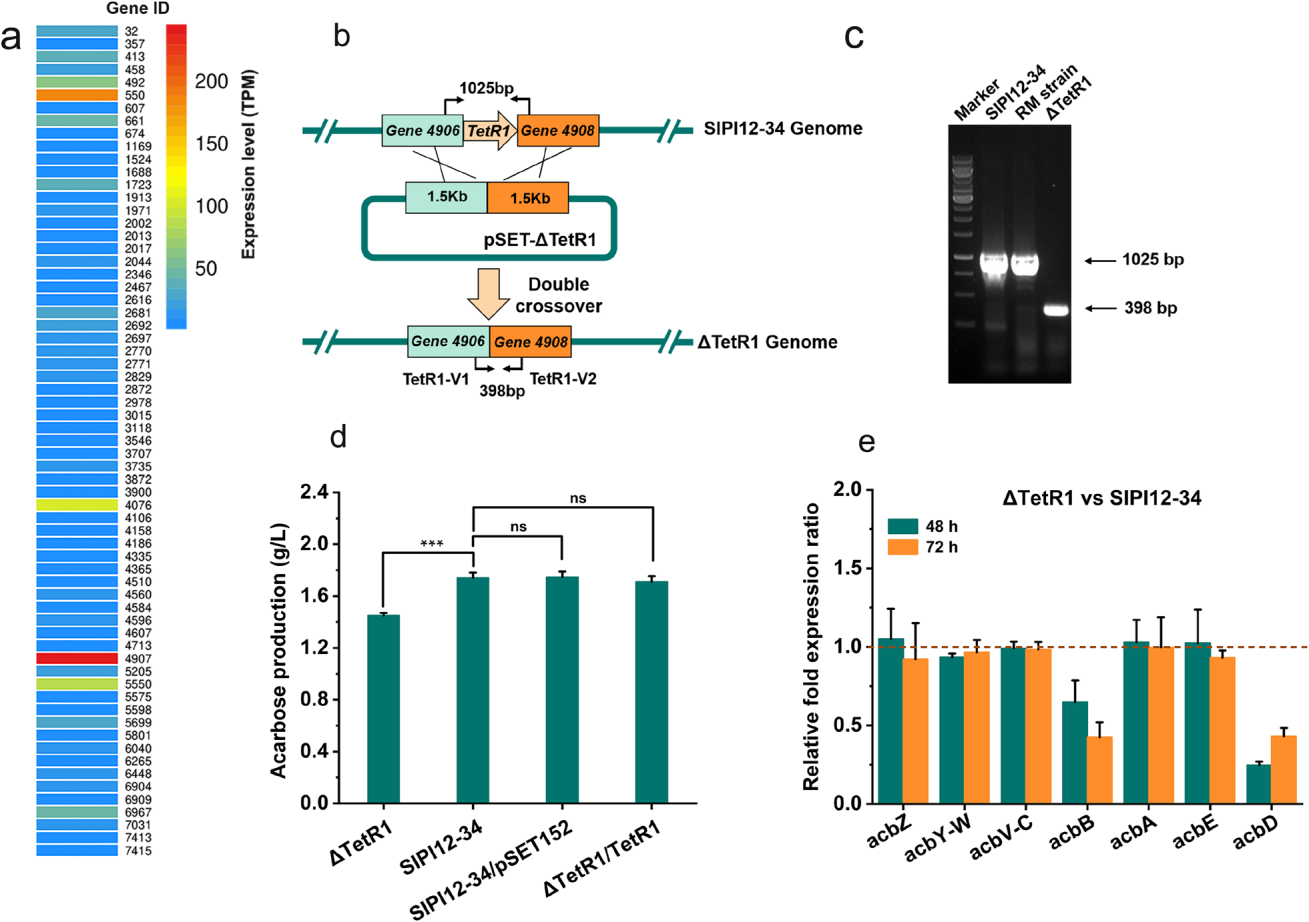
*TetR1* acts as a positive regulator of acarbose synthesis. **a** The expression of 65 TFRs was derived from transcriptome information. TPM (Transcripts Per Million reads): Number of read sections from a transcript of transcripts per million reading segments. **b** Schematic diagram of the strategy used for deletion of *TetR1* in SIPI12-34. TetR1-V1 and TetR1-V1 were primer pairs used for PCR validation and pSET-ΔTetR1 was knockout plasmid used for deletion of *TetR1*. **c** PCR validation of SIPI12-34, RM strain and ΔTetR1. RM strain: strain with no genome changes after double crossover recombination. ΔTetR1: mutant strain with deletion of *TetR1* (627 bp) in the chromosome of SIPI12-34. **d** Acarbose production profiles of SIPI12-34 and other mutant were analyzed by HPLC. ΔTetR1/TetR1: mutant strain with complement of *TetR1* under the control of native promoter. SIPI12-34/pSET152: SIPI12-34 with pSET152 plasmid acted as a control strain. Error bars show standard deviations, ***P < 0.001 ns: no significant. **e** Transcriptional analysis of acarbose BGC in SIPI12-34 and ΔTetR1 during the fermentation process by quantitative real-time PCR. RNA samples were isolated from SIPI12-34 and ΔTetR1 at 48 h and 72 h in shake flask culture. At each time point, the expression levels of each transcriptional unit in SIPI12-34 were taken as 1 to quantify the relative expression levels in ΔTetR1, respectively. Each strain has 3 replicates at different time points

TFRs are widely distributed among microorganisms; among them, actinomycetes have the largest number of members of this family. For example, *Streptomyces coelicolor* encodes 153 TFRs, *S. avermitilis* encodes 115 TFRs and *Saccharopolyspora erythraea* encodes 101 TFRs. TFRs are not only involved in regulating the primary metabolism of cells but they also regulate the metabolism of secondary metabolic products such as antibiotic biosynthesis and efflux [26, 27]. To investigate the effect of TFRs on SIPI12-34, we obtained an overview of its TFRs from the transcriptome information. A total of 65 genes in the genome were annotated as TetR family transcriptional regulators (Fig. 1a), and the transcriptome data indicated that most of them were expressed at very low or almost no expression levels; only *gene 4907* (*TetR1*) was expressed at a higher level. Reasonably, we deduced that *TetR1* has a stronger effect on primary metabolism or secondary metabolite production than the other TFRs.

To characterize *TetR1* in acarbose biosynthesis, the *TetR1* disruption mutant was constructed by a traditional method based on homologous double-crossover recombination (Fig. 1b). The mutant ΔTetR1 was obtained and confirmed by PCR (Fig. 1c). Subsequently, ΔTetR1 and the parent strain SIPI12-34 were cultivated, and their production of acarbose was quantified by HPLC analysis. As shown in Fig. 1d, the yield of acarbose in the ΔTetR1 mutant was decreased by 17% (1.45 g/L) in comparison to the parental strain SIPI12-34 (1.74 g/L). Then, the complementation of *TetR1* for ΔTetR1 was performed with the φC31-derived integrative plasmid pSET152 [28]. As expected, the yield of acarbose returned to 1.71 g/L, and SIPI12-34/pSET152 was used as the control strain (1.75 g/L). The dry cell weights of ΔTetR1 and SIPI12-34 at different times were measured, and the results indicated that ΔTetR1 and SIPI12-34 had similar growth levels (Additional file 1: Fig. S1). Taken together, the above findings confirmed that *TetR1* positively regulated the production of acarbose.

The acarbose BGC contains 22 genes divided into 7 transcriptional units [7, 22]. To investigate the effect of *TetR1* on acarbose BGC, the quantitative real-time PCR analysis of these seven transcriptional units of SIPI12-34 and ΔTetR1 was performed at 48 h and 72 h during fermentation. As shown in Fig. 1e, significantly lower expression levels of *acbB* and *acbD* were detected in ΔTetR1. At 48 h and 72 h, *acbB* decreased by 36% and 58%, respectively and *acbD* decreased by 76% and 57%, respectively. In the remaining five transcription units, no significant differences in expression levels were observed between SIPI12-34 and ΔTetR1. The *acbB* encoded dTDP-glucose-4,6-dehydratase in the synthesis pathway of the amino-deoxyhexose moiety. Acarbose yield improvement by intensifying the synthesis pathway of the amino-deoxyhexose moiety has been previously demonstrated [15]. In addition, our transcriptome data revealed that *acbB* had a relatively low expression level compared with the other genes from the acarbose BGC during the fermentation phase, which suggested a possible way to increase the yield by promoting *acbB* expression.

#### Overexpression of *TetR1* to increase acarbose production

**Fig. 2:**
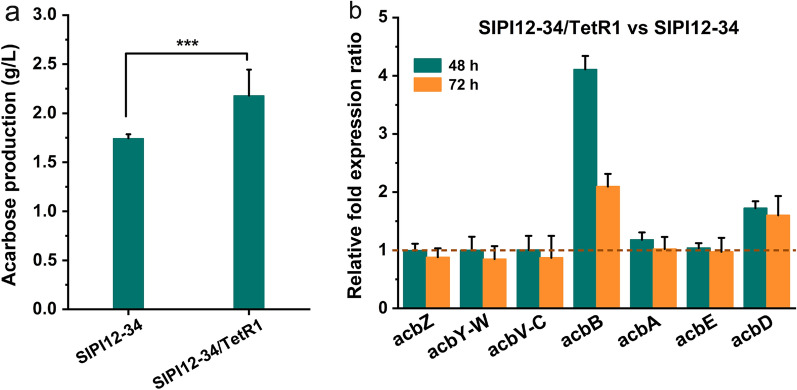
Overexpression of *TetR1* to increase acarbose production. **a** Acarbose production profiles of SIPI12-34 and SIPI12-34/TetR1 were analyzed by HPLC. SIPI12-34/TetR1: SIPI12-34 overexpressing the *TetR1*. Error bars show standard deviations, ***P < 0.001. **b** Transcriptional analysis of acarbose BGC in SIPI12-34 and SIPI12-34/TetR1 during the fermentation process by quantitative real-time PCR. RNA samples were isolated from SIPI12-34 and SIPI12-34/TetR1 at 48 h and 72 h respectively, in shake flask culture. At each time point, the expression levels of each transcriptional unit in SIPI12-34 were taken as 1 to quantify the relative expression levels in SIPI12-34/TetR1, respectively. Each strain has 3 replicates at different time points

*TetR1*, as a positive regulator of acarbose biosynthesis, was overexpressed as a reasonable way to increase the yield of acarbose. The SIPI12-34/TetR1 strain overexpressing *TetR1* in SIPI12-34 was constructed and cultured for 96 h in shake flask. As considered before, the productivity of SIPI12-34/TetR1 increased by approximately 25% (2.18 g/L) compared to that of the parent strain SIPI12-34 (Fig. 2a). Quantitative real-time PCR was carried out to investigate the expression of acarbose BGC at 48 h and 72 h, and the results indicated that the expression of *acbB* and *acbD* in SIPI12-34/TetR1 was significantly increased compared with that in SIPI12-34 (Fig. 2b). The remaining five transcriptional units did not change significantly. This suggested that the strategy of overexpressing *TetR1* to increase acarbose production was effective.

#### Overexpression of genes with low expression levels in acarbose BGC

**Fig. 3:**
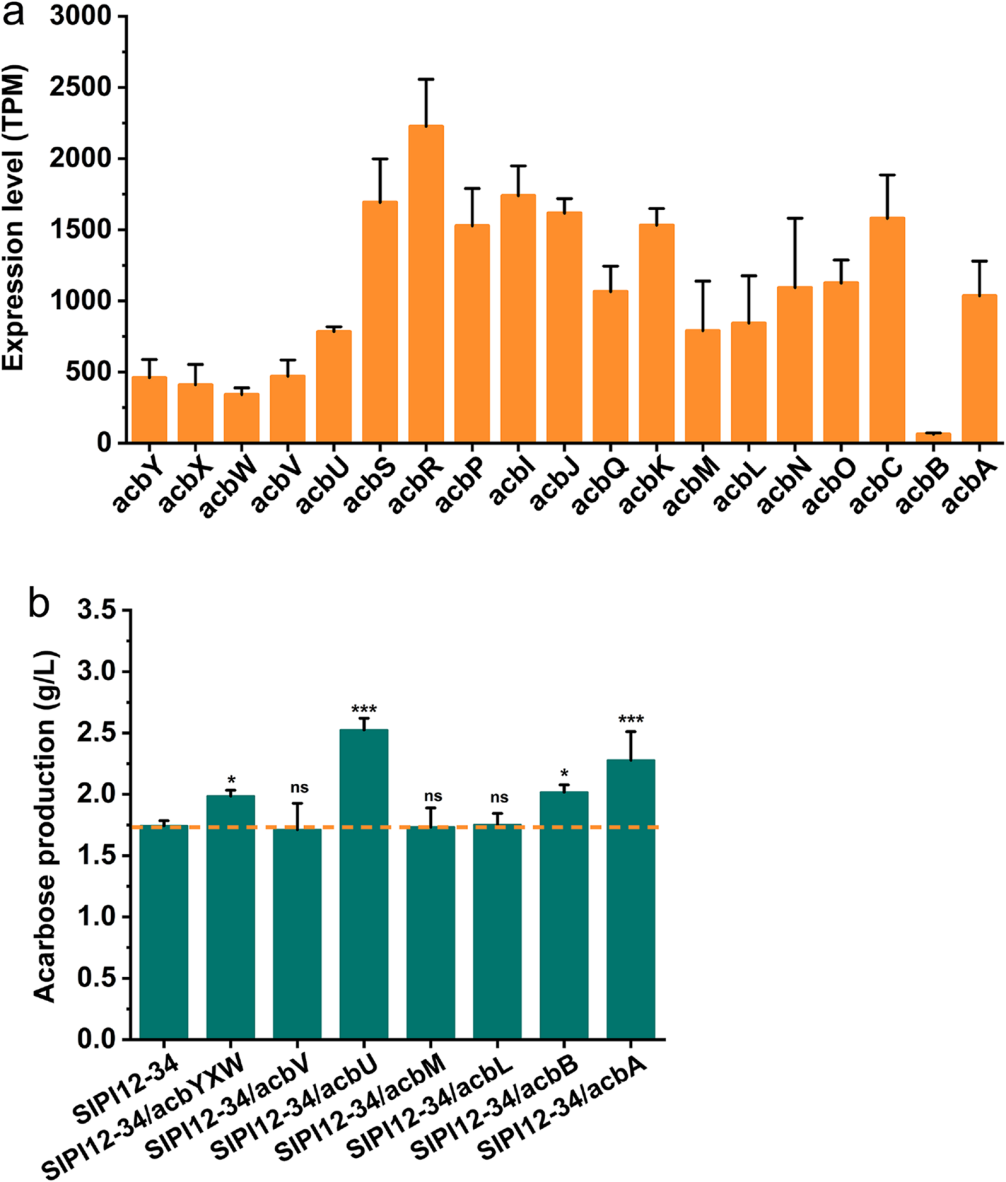
Overexpression of genes with low expression levels in acarbose BGC. **a** The expression of acarbose BGC derived from transcriptome sequencing. **b** Acarbose production profiles of SIPI12-34 and different genetic engineering strains obtained. Each mutant was obtained by performing an extra copy of the corresponding gene on SIPI12-34. The orange dotted line represents the yield of the parental strain. Error bars show standard deviations, ***P < 0.001, *P < 0.05 ns: no significant

Furthermore, according to the transcriptome information, we found that there were significant differences in the expression levels of the acarbose BGC. As shown in Fig. 3a, some genes were expressed at lower levels. We considered that these genes with lower expression might be restrictive factors in the process of acarbose synthesis and attempted to overexpress these genes [29], including *acbY*, *acbX*, *acbW*, *acbV*, *acbU*, *acbM*, *acbL* and *acbB*. In addition, *acbA* was also used as an overexpressed target gene to offset inefficient utilization of the amino-deoxyhexose synthesis pathway according to the guidance of previous research [15]. These genes were integrated into SIPI12-34 to construct overexpression strains. The acarbose production is shown in Fig. 3b. We found that the engineered strains overexpressing *acbYXW*, *acbU*, *acbA* and *acbB* achieved a more obvious production boost and the yield increased by approximately 14% (1.96 g/L), 45% (2.53 g/L), 30% (2.28 g/L) and 16% (2.02 g/L), respectively, compared with the parent strain SIPI12-34. However, overexpression of *acbV*, *acbM* and *acbL* did not lead to significant changes in yield. These genes were presumed not to be limiting factors in acarbose synthesis. The above experimental results showed that a feasible way to increase product yield is by overexpressing these low expression level genes.

### The pathway engineering strategies to enhance acarbose production

#### Down regulation of the glycolysis pathway

Most studies improved the production of desired products by increasing the content of precursors or adjusting the metabolic pathway to make the metabolic flux flow more to the target products [30]. Sedo-heptulose-7-phosphate and glucose-1-phosphate, as the two precursors of the first step in the acarbose synthesis pathway, are closely related to the formation of the final product. Glucose-1-phosphate is converted from glucose-6-phosphate catalyzed by phosphoglucomutase. In the cell, glucose-6-phosphate is transformed into pyruvate through the glycolysis pathway (EMP) and finally converted to CO_2_ and H_2_O with a large amount of ATP generation, which is inseparable from cell growth and metabolism. To allow more metabolic flux flow to the acarbose synthesis process, down regulation of the gene expression of key enzyme (6-phosphofructokinase) in EMP was applied by CRISPR-dcas9 system. However, the negative impact on the growth of cell resulted in a decrease in acarbose yield (Additional file 1: Fig. S4, S5). Then, we turned our attention to the regulation of glycogen metabolic pathways to elevate the amount of the precursor, glucose-1-phosphate.

#### Attenuation of glycogen synthesis and enhancement of glycogen degradation

**Fig. 4:**
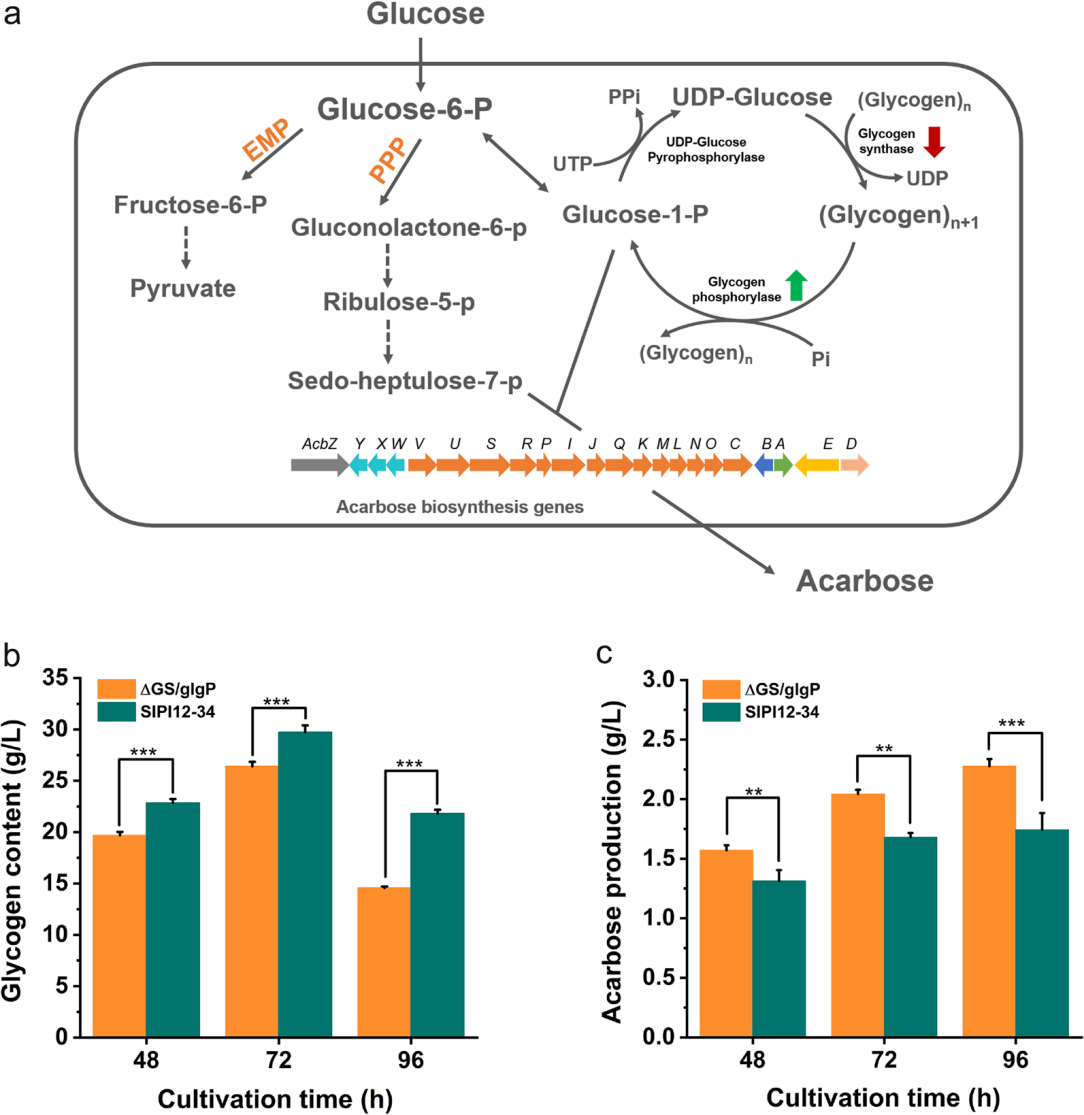
Attenuation of glycogen synthesis and enhancement of glycogen degradation. **a** Overview illustration of the metabolic pathways involved in the two precursors in the synthesis of acarbose. The red arrow down represents the weakened glycogen synthesis pathway, and the green arrow up represents the enhanced glycogen degradation pathway. **b** Determination of glycogen content in SIPI12-34 and ΔGS/glgP during the fermentation process. **c** Acarbose production profiles of SIPI12-34 and ΔGS/glgP were analyzed by HPLC. Error bars show standard deviations, ***P < 0.001, **P < 0.01

As shown in Fig. 4a, the two key rate-limiting enzymes in glycogen metabolism are glycogen synthase and glycogen phosphorylase. Weakening glycogen synthesis and strengthening glycogen degradation were adopted to reduce glucose-1-phosphate flow to the glycogen metabolic pathways. According to the transcriptome data, six genes were annotated as glycogen synthase, they were *gene 2160*, *gene 2664*, *gene 3312*, *gene 4463*, *gene 4472* and *gene 6987*, respectively. And *glgP* was annotated as glycogen phosphorylase. Among the six genes encoding glycogen synthase, only *gene 6987* was at a relatively high expression level (Additional file 1: Fig. S6). Hence, we first attempted to delete *gene 6987* from the genome of SIPI12-34 to reduce the amount of glycogen synthesis. *Gene 6987* was completely deleted to obtain the mutant strain ΔGS (Additional file 1: Fig. S7). Next, a cloned *glgP* with a strong promoter PermE* was integrated into ΔGS to create the mutant ΔGS/glgP, aiming at strengthening the glycogen degradation pathway so that more glycogen was converted to glucose-1-phosphate. Then, SIPI12-34 and mutant ΔGS/glgP were cultivated in fermentation medium for 96 h, and fermentation broth samples were extracted at 48 h, 72 h, and 96 h to determine the glycogen content and acarbose production. The anthrone chromogenic assay was used to quantify the glycogen content by UV spectrophotometer. As shown in Fig. 4b, we observed that the glycogen content of ΔGS/glgP was decreased at all three time points compared with SIPI12-34, and at 96 h it had decreased by approximately 33%. As predicted, there was an associated increase in acarbose production, increasing 31% to 2.27 g/L at 96 h (Fig. 4c). This suggested that the glycogen content was reduced due to the regulation of the glycogen metabolic pathways, and more glucose-1-phosphate was retained for acarbose synthesis. In addition, the determination of cell dry weights showed that SIPI12-34 and ΔGS/glgP had similar biomass levels (Additional file 1: Fig. S8), indicating that the regulation of the glycogen metabolism pathway did not have a significant effect on cell growth.

### Combination of the effective engineering strategies

**Fig. 5:**
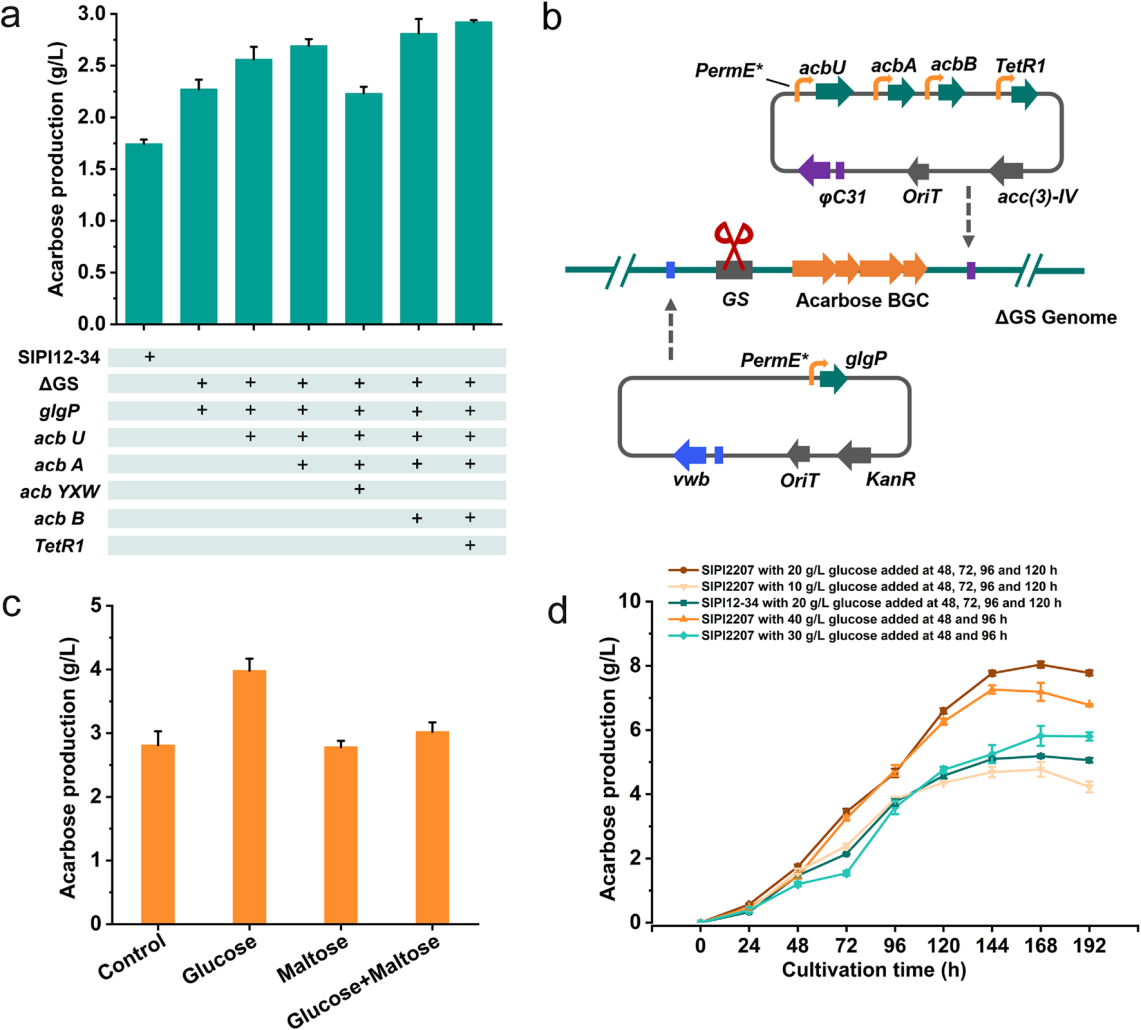
Combination of the effective engineering strategies and development of a fed-batch fermentation strategy. **a** Acarbose production profiles of SIPI12-34 and other mutants combined different genes overexpression were analyzed by HPLC. The table below illustrates the different combination strategies. Error bars show standard deviations. **b** Schematic diagram of genetic engineering for obtaining SIPI2207. *GS* represents the gene encodes glycogen synthase. **c** Acarbose production of SIPI2207 with addition of different sugars during the fermentation process. Control represents no supplementation for SIPI2207. Glucose represents supplementation of 20 g/L glucose for SIPI2207 at 48 h. Maltose represents supplementation of 20 g/L maltose for SIPI2207 at 48 h. Glucose + Maltose represents supplementation of a 10 g/L mixture of glucose and maltose for SIPI2207 at 48 h. **d** Detection of acarbose production of SIPI2207 and SIPI12-34 with addition of different amounts of glucose at different culture time

With overexpression of the above genes and regulation of glycogen metabolic pathways, the production of acarbose was improved to varying degrees; therefore, the combination of these strategies was carried out to see if there was an even greater improvement. First, ΔGS/glgP served as the starting strain to perform overexpression, and the introduction of pSET152-acbU resulted in a yield increase from 2.27 g/L to 2.56 g/L (Fig. 5a). Subsequently, pSET152-acbU + acbA was constructed and introduced into GS/glgP to continue the multigene overexpression, and the yield further increased to 2.71 g/L. Unexpectedly, the next introduction of *acbYXW* caused an 18% decrease in yield to 2.23 g/L. Instead of *acbYXW*, we imported pSET152-acbU + acbA + acbB into GS/glgP, and the yield returned to a higher level of 2.81 g/L. Eventually, integration of the regulator *TetR1* created the engineered strain ΔGS/glgP + acbU + acbA + acbB + TetR1 (SIPI2207) with the highest yield up to 2.92 g/L, and the process of multigene overexpression is shown in Fig. 5b. After several rounds of gene combination, the yield of the engineered strain SIPI2207 increased by 68% compared with that of the parent strain SIPI12-34. In addition, the detection of dry cells weight showed that the integration of multiple genes in the strain had no obvious effect on cells growth (Additional file 1: Fig. S9).

### Development of a fed-batch fermentation strategy

Fed-batch glucose and maltose have been demonstrated to increase the acarbose yield effectively [31]. To further enhance the production capacity of SIPI2207, glucose, maltose and a mixture of glucose and maltose were added to the medium at 48 h, respectively. After 96 h of fermentation, we found that the addition of glucose resulted in a more pronounced increase in yield (3.98 g/L), while the addition of maltose had little effect (Fig. 5c). The detection of the dry cell weight indicated that the addition of glucose significantly increased the amount of cell (Additional file 1: Fig. S10). Subsequently, to find a more effective sugar supplementation condition, different concentrations of glucose were added at different culture time to investigate the impact on acarbose production. As shown in Fig. 5d, addition of glucose 40 g/L at 48 h and 96 h; addition of glucose 30 g/L at 48 h and 96 h; addition of glucose 20 g/L at 48 h, 72 h, 96 h and 120 h; and addition of glucose 10 g/L at 48 h, 72 h, 96 h and 120 h were adopted, respectively. With the addition of extra glucose, the fermentation time was significantly prolonged. Among these different feeding strategies, the highest yield of acarbose in SIPI2207 reached 8.04 g/L, with 20 g/L glucose added at 48 h, 72 h, 96 h and 120 h. Similarly, the yield of SIPI12-34 was significantly increased to 5.10 g/L under this fermentation condition. This suggested that the addition of glucose during the fermentation process was beneficial to prolong the fermentation time and increase the production of acarbose. It is worth noting that the engineered strain SIPI2207 still produced 58% more acarbose than SIPI12-34 with the same fed-batch culture strategy.

## Discussion

Random mutagenesis and media optimization to improve productivity have been widely applied in industrial production [32, 33], but these traditional methods are laborious, and thus genetic engineering has received increasing attention. With insufficient knowledge about the regulatory mechanisms of acarbose synthesis and metabolism, rational strain design to improve the production of acarbose is restricted. In streptomyces, it is common for TFRs to be the target of genetic engineering to regulate the synthesis of secondary metabolites. AtrA and DepR1, two TFRs in *S. roseosporus*, were shown to positively regulate the expression of daptomycin BGC by binding to the *dptE* promoter [34, 35]. Based on the positive effects, overexpression of both genes by genetic engineering increased daptomycin production. In addition, some TFRs affect the synthesis of final products by regulating the expression of genes outside the cluster. Zhen identified a TFR (*pccD*) that had a negative effect on the synthesis of abamectin by downregulating the transcriptional level of propionyl-CoA carboxylase in *Saccharopolyspora erythraea* [36]. Liu proved that a TFR (SAV7471) negatively regulated erythromycin synthesis by reducing the supply of propionyl-CoA in *S. avermitilis* [37]. In our study, a TFR (*TetR1*) was discovered and screened through the analysis of transcriptome information. Real-time quantitative PCR analysis of SIPI12-34 and ΔTetR1 indicated that *TetR1* was involved in promoting the expression of *acbB* and *acbD*. The *acbB* is involved in the synthesis pathway of the amino-deoxyhexose moiety and an increase in its expression level directly affects the formation of acarbose. *AcbD*, which encodes an extracellular glycosyltransferase, is not directly involved in acarbose biosynthesis [38]. We have also tried to overexpress *acbD* in SIPI12-34, but the strain showed almost the same productivity as the parental strain (Additional file 1: Fig. S11). In addition to being regulated by *TetR1*, *acbD* is also regulated by *AcrC* [22] and has been speculated to influence acarbose synthesis through other indirect effects such as feedback inhibition or have additional enzymatic functions. These inferences required us to do more work for further verification.

Metabolic pathway optimization by genetic engineering plays an important role in the construction of engineered strains. In this study, the regulation of glycogen metabolic pathways caused a great improvement in the production of the final products. Glucose-1-phosphate, as a precursor in the synthesis of acarbose, is derived from glucose-6-phosphate. While glucose-6-phosphateas is an important intermediate mainly involved in the EMP, we suppressed the expression of the key gene in EMP through the CRISPR-dcas9 system [39], expecting more precursors to be available for use in the synthesis of acarbose. Unfortunately, cell growth was greatly negatively affected, determination of dry cell weights showed a 17% decrease at 96 h, and the yield decreased 19% compared to the parental strain. There is an important balance between primary metabolic pathways and secondary metabolic pathways, and it is necessary to adjust this balance to obtain more of the target products. Tian designed a novel quorum-sensing system with CRISPRi in *S. rapamycinicus* to realize dynamic regulation and boosted the rapamycin titer approximately 6.6-fold [40]. The application of this system did not affect cell growth and significantly improved the production of rapamycin, and it addressed the metabolic imbalances caused by static engineering. To further enhance the production of acarbose, we could also try to adjust the balance between primary metabolism and acarbose synthesis through dynamic regulation.

The addition of S-adenosylmethionine [41] and validamine [42] to the culture and fermentation at an elevated osmolality [43] had been demonstrated to promote the production of acarbose. In addition, a series of optimizations were made to the fermentation process by constructing a genome-scale metabolic model of *Actinoplanes* sp. SE50/110 [44]. In our study, we attempted to increase the yield by feeding glucose and maltose during fermentation, and the results showed that supplementation with extra glucose was more effective for maintaining cells growth and improve the acarbose production. In addition, the amount and time of supplemental glucose had been further optimized to obtain a more suitable culture condition. With the fed-batch strategy, the engineered strain SIPI2207 made a breakthrough in acarbose yield and reached the highest titer ever reported (Table 1). Julian Droste found that the transcription level of acarbose synthesis genes showed a continuously decreasing trend from the early growth stage to the stationary stage, and acarbose was highly produced during the early growth stage and its production ceased during the stationary stage [45]. This finding also gave us some guidance by extending the growth phase can be an effective strategy for enhancing the production of acarbose.

**Table 1:** Comparison of genetic engineering strategies and yield in acarbose producing strain

Strain	Strategy	Reported production (g/L)	References
*Actinoplanes* sp. SIPI12-34	Combination of multiple strategies and fed-batch fermentation	8.04	This study
*Actinoplanes* sp. SE50/110	Minimized the flux to the shunt products and maximized the supply of the amino-deoxyhexose moiety	7.4	[15]
*Actinoplanes* sp. SE50/110	Deletion of *ACWT_4325* and *ACWT_7629*	4.21	[25]
*Actinoplanes* sp. SE50/110	Introduction of an additional acarbose gene cluster	3.18	[23]

## Conclusions

In this study, *TetR1* was screened and identified as a positive regulator to the synthesis of acarbose. Moreover, through overexpression of *TetR1* and the genes with low expression level in acarbose BGC, combined with the regulation of glycogen metabolism pathways and optimization of culture conditions, the production of acarbose in the engineered strain was significantly improved.

## Methods

### Chemicals and reagents

All DNA polymerase, T4 DNA ligase and reverse transcriptase were purchased from Takara Biomedical Technology. All restriction enzymes used in this study were purchased from Thermo Fisher Scientific. The Glycogen Content Assay Kit and the other molecular biology kits were purchased from Sangon Biotech.

### Strains, plasmids, primers, and culture conditions

All strains and plasmids used in this study are listed in Additional file 2: Table S1, and all primers used are listed in Additional file 3: Table S2. The original strain SIPI12-34 was obtained from *Actinoplanes* sp. SE50/110 through multiple rounds of physical and chemical mutagenesis and natural screening.

Acarbose was obtained by fermentation, the SIPI12-34 was diluted and spread on solid medium (3% soluble starch, 2% glucose, 1% peptone, 0.1% yeast extract, 2% soy flour, 0.1% CaCO_3_, 2% agar, pH = 7) and cultured at 28 °C for approximately 7 days. A single colony was picked and transferred to 25 ml seed medium (1% soluble starch, 2% glycerin, 1% glucose, 3% soy flour, 0.2% CaCO_3_, pH = 7) and cultured at 28 °C, 200 rpm for 3 days in shaking flask, and then 10% of the inoculum was transferred to 25 ml fermentation medium (3% glucose, 3% maltose, 3% soy flour, 0.4% K_2_HPO_4_·3H_2_O, 0.15% FeCl_3_·6H_2_O, 0.35% CaCl_2_, 0.3% CaCO_3_, pH = 7.2) and cultured at 28 °C, 200 rpm for 4 days in shaking flask.

*E. coli* strains were cultured on LB agar plates or in LB liquid medium at 37 °C. *E. coli* DH5α was used for gene cloning, and *E. coli* ET12567 and S17 were used for the intergeneric conjugation between *E. coli* and mycelia of *Actinoplanes* sp. For intergeneric conjugation, SIPI12-34 and *E. coli* ET12567 or S17 were cultured in TSB liquid (3% TSB) medium and LB (1% NaCl, 1% peptone, 0.5% yeast extract) liquid medium at 28 °C and 37 °C, respectively. Their mixtures were incubated on MS solid medium (2% mannitol, 2% soy flour, 2% agar) at 28 °C for 5 days. During this period, apramycin (0.04 mg/ml) and trimethoprim (0.04 mg/ml) need to be added to the MS solid medium for selection.

### Construction of gene knockout strains

The knockout strains ΔTetR1 and ΔGS were derived from SIPI12-34 with *gene 4076* (627 bp) and *gene 6987* (2073 bp) completely deleted, respectively. Taking the construction of the ΔTetR1 mutant as an example, it was obtained through a traditional method based on homologous recombination. The two homologous arms were amplified from SIPI12-34 genomic DNA using the primer pairs TetR1-UP-F/R and TetR1-DOWN-F/R. Then, they were ligated together by overlapping PCR using the primer pairs TetR1-UP-F and TetR1-DOWN-R. The final PCR product was digested by *Hin*dIII/*Xba*I and assembled into the pSET vector to obtain the knockout plasmid pSET-ΔTetR1. It was introduced into *E. coli* DH5α for plasmid cloning. After sequencing verification, it was transferred to SIPI12-34 using conjugation with the *E. coli* ET12567 strain as a host. Then, the first crossover mutants were obtained by apramycin screening and they were sub-cultured in TSB liquid to obtain the second crossover mutants. Subcultures were conducted 6 times. The second crossover mutants were screened by PCR using the primer pairs TetR1-V1/V2 for verification. With the deletion of a 627 bp fragment on the genome, the PCR product for the correct strain appeared as a band of 398 bp. The PCR product of ΔTetR1 was sequenced by Sangon Biotech for verification. All primers used were synthesized by Jie Li Biology.

The method of construction of the mutant ΔGS was the same as ΔTetR1, and the two homologous arms were amplified from SIPI12-34 genomic DNA using the primer pairs GS-UP-F/R and GS-DOWN-F/R. The primer pairs used in the final PCR verification were GS-V1 and GS-V2. With the deletion of a 2073 bp fragment on the genome, the PCR product for the correct strain appeared as a band of 524 bp. The PCR product of ΔGS was sequenced by Sangon Biotech for verification.

### Gene complement and overexpression

We selected pSET152 and pSET155 as the vectors used for gene complementation and overexpression, since they can shuttle between *Actinomycetes* and *E. coli* and have a strong promoter, PermE*. The plasmid pSET152-TetR1 was constructed for the *TetR1* complement of ΔTetR1. *TetR1* was amplified from SIPI12-34 genomic DNA using the primer pairs TetR1-F/TetR1-R, and then the PCR product was inserted between *Xba*I and *Sgs*I in the pSET152 vector. The vector pSET152-TetR1 replaced the strong promoter PermE* with a native promoter. After sequencing by Sangon Biotech for verification, it was transferred ΔTetR1 to using conjugation with the *E. coli* ET12567 strain as a host to obtain the mutant ΔTetR1/TetR1. Apramycin and trimethoprim were added during cultivation to select the correct strain. As controls, the empty vector pSET152 was transferred into SIPI12-34 to obtain SIPI12-34/pSET152.

For overexpression of *TetR1*, it was amplified by the primer pairs TetR-F and TetR-R. Then, the PCR product was assembled into pSET152 between *Nde*I and *Sgs*I to obtain pSET152-TetR. Strategies for overexpression of the other genes are as follows: *glgP*, *acbYXW*, *acbV*, *acbU*, *acbM*, *acbL*, *acbB*, *acbA* and *acbD* were amplified by the primer pairs glgP-F/R, acbYXW-F/R, acbV-F/R, acbU-F/R, acbM-F/R, acbL-F/R, acbB-F/R, acbA-F/R and acbD-F/R, respectively. These PCR products were assembled into pSET155 or pSET152 between *Nde*I and *Sgs*I to obtain pSET155-glgP, pSET152-acbYXW, pSET152-acbV, pSET152-acbU, pSET152-acbM, pSET152-acbL, pSET152-acbB, pSET152-acbA and pSET152-acbD. After sequencing and verification, they were introduced into SIPI12-34 or ΔGS to form the corresponding mutants.

To further improve the acarbose yield, we combined the genes that had a positive effect on yield in our study. The PermE* + *acbA* fragment was amplified using the primer pair UA-F/R and assembled into pSET152-acbU between *Eco*RV and *Pac*I to obtain pSET152-acbU + acbA. The PermE* + *acbB* fragment was amplified using the primer pairs AB-F/R and assembled into pSET152-acbU + acbA between *Pac*I and *Spe*I to obtain pSET152-acbU + acbA + acbB. Analogously, the PermE* + *acbYXW* fragment was amplified using the primer pairs AY-F/R and assembled into pSET152-acbU + acbA between *Pac*I and *Spe*I to obtain pSET152-acbU + acbA + acbYXW. The PermE* + *TetR1* fragment was amplified using the primer pair BT-F/R and assembled into pSET152-acbU + acbA + acbB between *Spe*I and *Pci*I to obtain pSET152-acbU + acbA + acbB + TetR1.

### Down regulation of the gene expression by CRISPR-dcas9 system

To investigate the effect of inhibiting the EMP on acarbose production and cell growth, the genes encoding 6-phosphofructokinase was targeted for gene suppression. The sgRNA was designed and amplified by the primer pairs dcas9-EMP-F/dcas9-R and then assembled into pSET-dcas9 between *Eco*RI and *Spe*I to obtain pSET-dcas9-EMP. After sequencing and verification, it was introduced into SIPI12-34 to obtain the corresponding mutant.

### RNA isolation and RNA-seq

Total RNA was extracted from mycelium using TRIzol reagent according to the manufacturer's instructions, and we used DNase I (Takara) to remove the genomic DNA. The RNA quality was then determined by a 2100 Bioanalyzer (Agilent) and quantified by ND-2000 (NanoDrop Technologies). High-quality RNA was used for subsequent quantitative real-time PCR and RNA-seq (Additional file 1: Fig. S2, Fig. S3). The RNA-seq was performed by Majorbio (Shanghai, China). The extracted RNA sample was used for cDNA library construction. RNA samples were subjected to rRNA depletion using the Ribo-Zero kit according to the manufacturer's instructions. The mRNAs were fragmented into short fragments (200-nt) by adding fragmentation buffer. The first-strand cDNA was produced by random hexamer-primed reverse transcription, followed by the synthesis of the second-strand cDNA using RNase H and DNA polymerase I.Then the fragments of cDNA were purified using the QIA quick PCR extraction kit. The cDNA library sequencing was performed on the Illumina sequencing platform (Illumina HiSeq™ 2000, Illumina, San Diego, CA, USA). Annotation of functional gene was conducted by comparison to the Swiss-prot, NR, Pfam, COG and GO databases.

### Analysis of the transcriptional levels by quantitative real-time PCR

The expression levels of seven transcriptional units in the acarbose synthesis gene cluster between ΔTetR1, SIPI12-34/TetR1 and SIPI12-34 were quantified by quantitative real-time PCR, and the experimental steps were performed according to the method reported by Dun et al. [46]. We collected the fermentation extracts at 48 h, and 72 h during the fermentation phase and extracted RNA as described above. Three samples were taken at each time point, and PCR was conducted in triplicate for each tested gene. We regarded the 16S rRNA gene as an internal reference gene to normalize the transcript level in the quantitative real-time PCR analysis. All primers used are listed in Additional file 3: Table S2.

### HPLC analysis of acarbose production

The supernatant of the fermentation broth was obtained by centrifugation at 12,000 rpm for 10 min and diluted 5 times using the mobile phase. The mobile phase consisted of 60% acetonitrile and 40% phosphate buffer, which contained 0.9 g of KH_2_PO_4_ and 0.25 g of Na_2_HPO_4_ per liter. The yield of acarbose was quantified by HPLC analysis with an Ultimate Hilic-NH2 column (4.6 × 250 mm, 5 μm) at 35 °C. The flow rate was 1.0 ml/min, and the wavelength was 210 nm. Each strain had at least 6 replicates.

### Analysis of glycogen content

To measure the glycogen content of SIPI12-34 and ΔGS/glgP, we extracted glycogen according to the instructions of the glycogen content assay kit. The samples were collected from the fermentation medium at different time points, the supernatant was discarded by centrifugation at 12,000 rpm, and then the cells were disrupted by sonication. The disrupted cells were incubated in a boiling water bath for 20 min, and after cooling, freshly prepared anthrone/H_2_SO_4_ solution was added and incubated in a boiling water bath for 15 min. Then, it was detected by UV spectrophotometry at 620 nm. The detection of glycogen content was performed by a standard curve using a gradient concentration of glucose solution. A total of 111 µg of glucose with an anthrone reagent is the same color as 100 µg of glycogen with an anthrone reagent. Both strains at every time point had 3 replicates.

### Determination of the cell dry weight

Four milliliters of fermentation culture was collected and centrifuged at 12,000 rpm for 10 min and washed with 95% NaCl solution. Then, the samples were dried at 55 °C in an oven until a constant weight was reached. Each group of cells weighed had three replicates.

### Statistical analysis

Each experiment had at least three replications, with the error bars showing the standard deviations. To compare differences between the test and the control data, P values were calculated by Student’s t-test.

## Supplementary Information

**Additional file 1: Figure S1.** Dry cell weights of SIPI12-34, ΔTetR1 and ΔTetR1/TetR1 at different times during the fermentation stage. **Figure S2.** The elimination of genomic DNA in obtained RNA of SIPI12-34 and ΔTetR1 were confirmed by PCR amplification using the primer pairs 16S1-F/R. **Figure S3.** The elimination of genomic DNA in obtained RNA of SIPI12-34 and SIPI12-34/TetR1 were confirmed by PCR amplification using the primer pairs 16S1-F/R. **Figure S4.** Acarbose production profiles of SIPI12-34 and SIPI12-34/dCas9-EMP were analyzed by HPLC. **Figure S5.** Dry cell weights of SIPI12-34 and SIPI12-34/dCas9-EMP at different times during the fermentation stage. **Figure S6.** The expression of genes encoding glycogen synthase derived from transcriptome sequencing. **Figure S7.** PCR validation of ΔGS and SIPI12-34. **Figure S8.** Dry cell weights of SIPI12-34 and ΔGS/glgP at different times during the fermentation stage. **Figure S9.** Dry cell weights of SIPI12-34 and SIPI2207 at different times during the fermentation stage. **Figure S10.** Dry cell weights of SIPI2207 with addition of different sugars during the fermentation stage. **Figure S11.** Acarbose production profiles of SIPI12-34 and SIPI12-34/acbD were analyzed by HPLC.

**Additional file 2: Table S1.** Strains and plasmids used in this study.

**Additional file 3: Table S2.** Primers used in this study.

## Data Availability

All data generated or analyzed during this study are included in this article and its Additional file.

## Abbreviations

BGC: Biosynthetic gene cluster
TFR: TetR family transcriptional regulator
*S.*: *Streptomyces*
EMP: Glycolysis pathway
