# Supplementary material for: Enhancement of acarbose production by genetic engineering and fed-batch fermentation strategy in Actinoplanes sp. SIPI12-34
Source: Microb Cell Fact. 2022 Nov 23;21:240. doi: 10.1186/s12934-022-01969-0 (PMC9685945; doi:10.1186/s12934-022-01969-0)
Supplement: Supplementary file 1 — Additional file 1: Figure S1. Dry cell weights of SIPI12-34, ΔTetR1 and ΔTetR1/TetR1 at different times during the fermentation stage. Figure S2. The elimination of genomic DNA in obtained RNA of SIPI12-34 and ΔTetR1 were confirmed by PCR amplification using the primer pairs 16S1-F/R. Figure S3. The elimination of genomic DNA in obtained RNA of SIPI12-34 and SIPI12-34/TetR1 were confirmed by PCR amplification using the primer pairs 16S1-F/R. Figure S4. Acarbose production profiles of SIPI12-34 and SIPI12-34/dCas9-EMP were analyzed by HPLC. Figure S5. Dry cell weights of SIPI12-34 and SIPI12-34/dCas9-EMP at different times during the fermentation stage. Figure S6. The expression of genes encoding glycogen synthase derived from transcriptome sequencing. Figure S7. PCR validation of ΔGS and SIPI12-34. Figure S8. Dry cell weights of SIPI12-34 and ΔGS/glgP at different times during the fermentation stage. Figure S9. Dry cell weights of SIPI12-34 and SIPI2207 at different times during the fermentation stage. Figure S10. Dry cell weights of SIPI2207 with addition of different sugars during the fermentation stage. Figure S11. Acarbose production profiles of SIPI12-34 and SIPI12-34/acbD were analyzed by HPLC. [file 12934_2022_1969_MOESM1_ESM.docx]

Supplementary information (Fig. S1-S11)

**Enhancement of acarbose production by** **genetic engineering and fed-batch fermentation strategy in** ***Actinoplanes* sp. SIPI12-34**

Zhenxin Li^1^, Songbai Yang^1^, Zhengyu Zhang^1,2^, Yuanjie Wu^1^, Jiawei Tang^1^, Luoju Wang^3^ and Shaoxin Chen^1*^

^1^ State Key Laboratory of New Drug and Pharmaceutical Process, China State Institute of Pharmaceutical Industry, Shanghai Institute of Pharmaceutical Industry, Shanghai 201203, China

^2^ Department of Biological Medicines & Shanghai Engineering Research Center of Immunotherapeutics, School of Pharmacy, Fudan University, Shanghai 201203, China

^3^ Shandong Qilu King-Pharmaceutical Co., Ltd. No.21 Qinglong Road, Pingyin County, Jinan, China


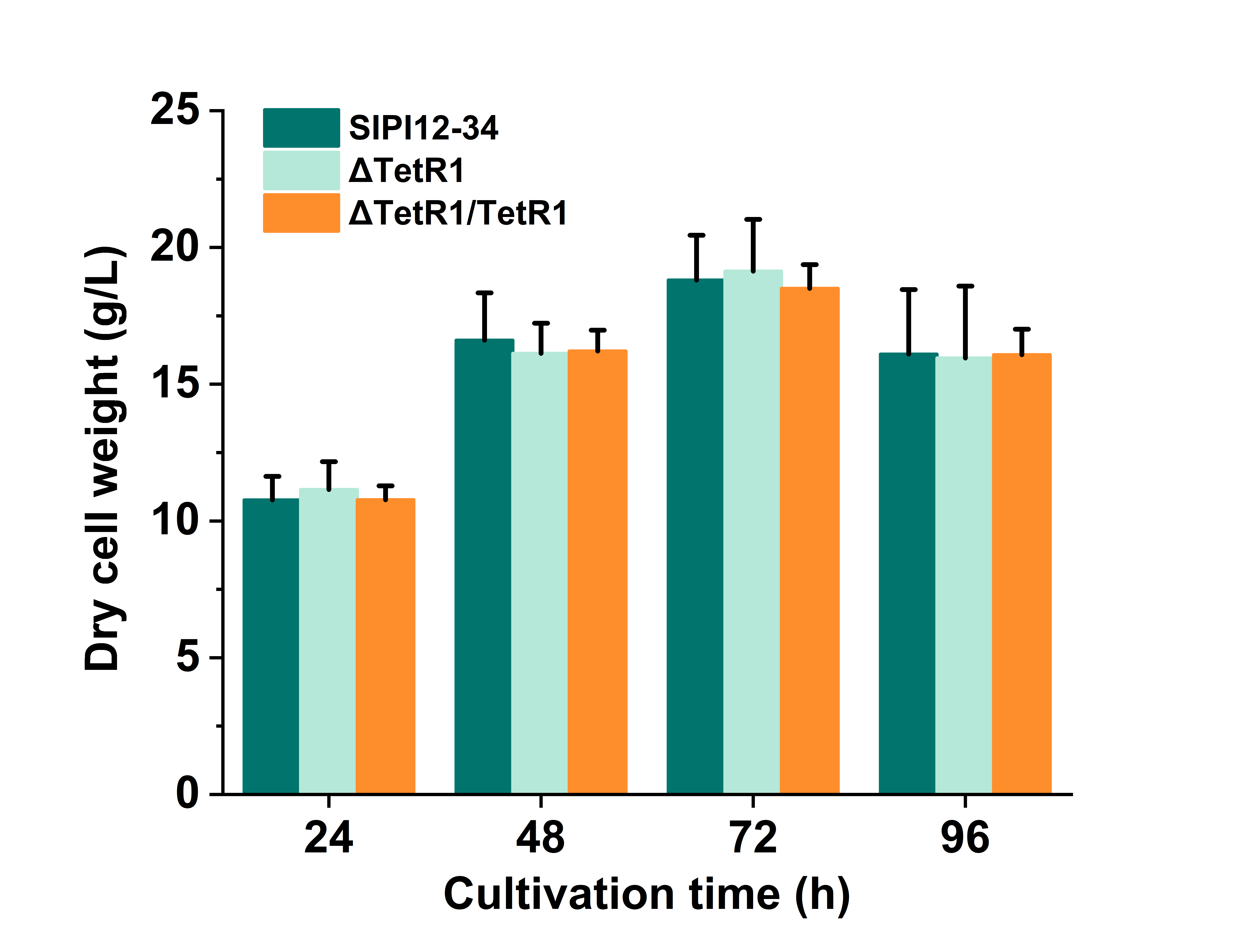


**Fig. S1** Dry cell weights of SIPI12-34, ΔTetR1 and ΔTetR1/TetR1 at different times during the fermentation stage. Error bars show standard deviations, three replicates for each strain at each time point.


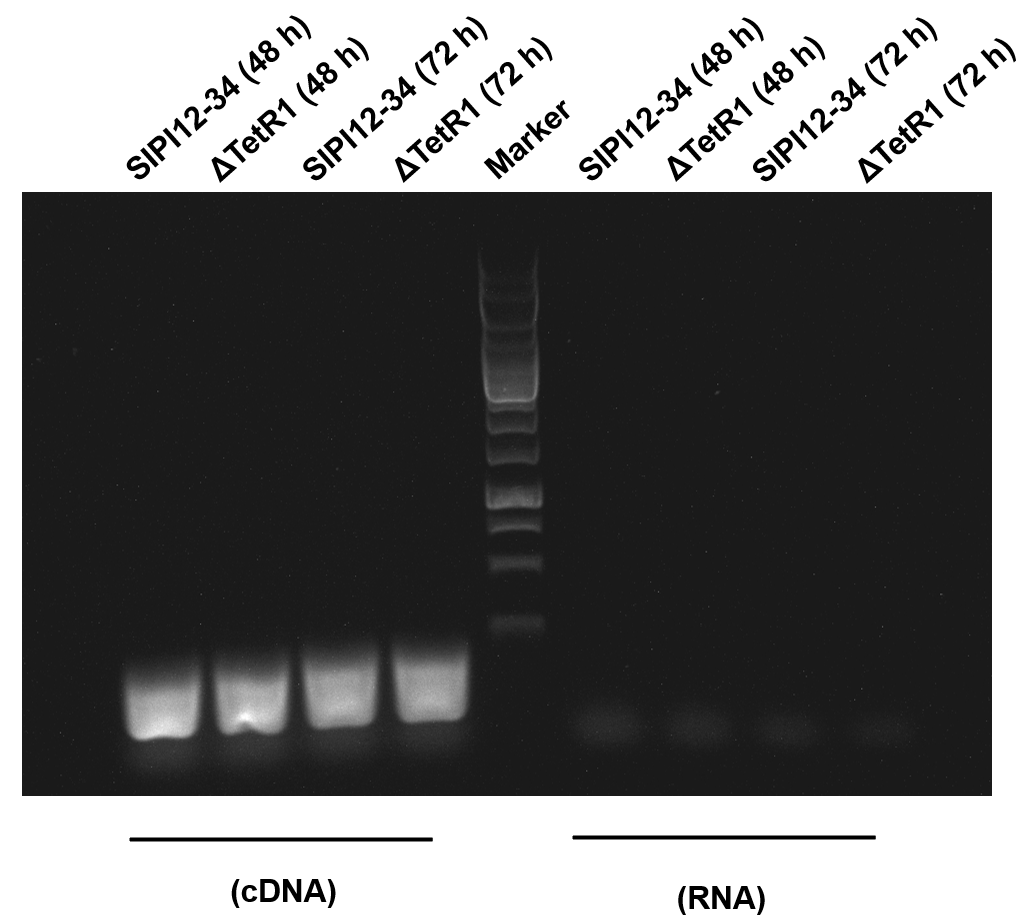


**Fig. S2** The elimination of genomic DNA in obtained RNA of SIPI12-34 and ΔTetR1 were confirmed by PCR amplification using the primer pairs 16S1-F/R. In PCR validation, there was no band display using RNA as template, and 200 bp bands were obtained using cDNA as template.


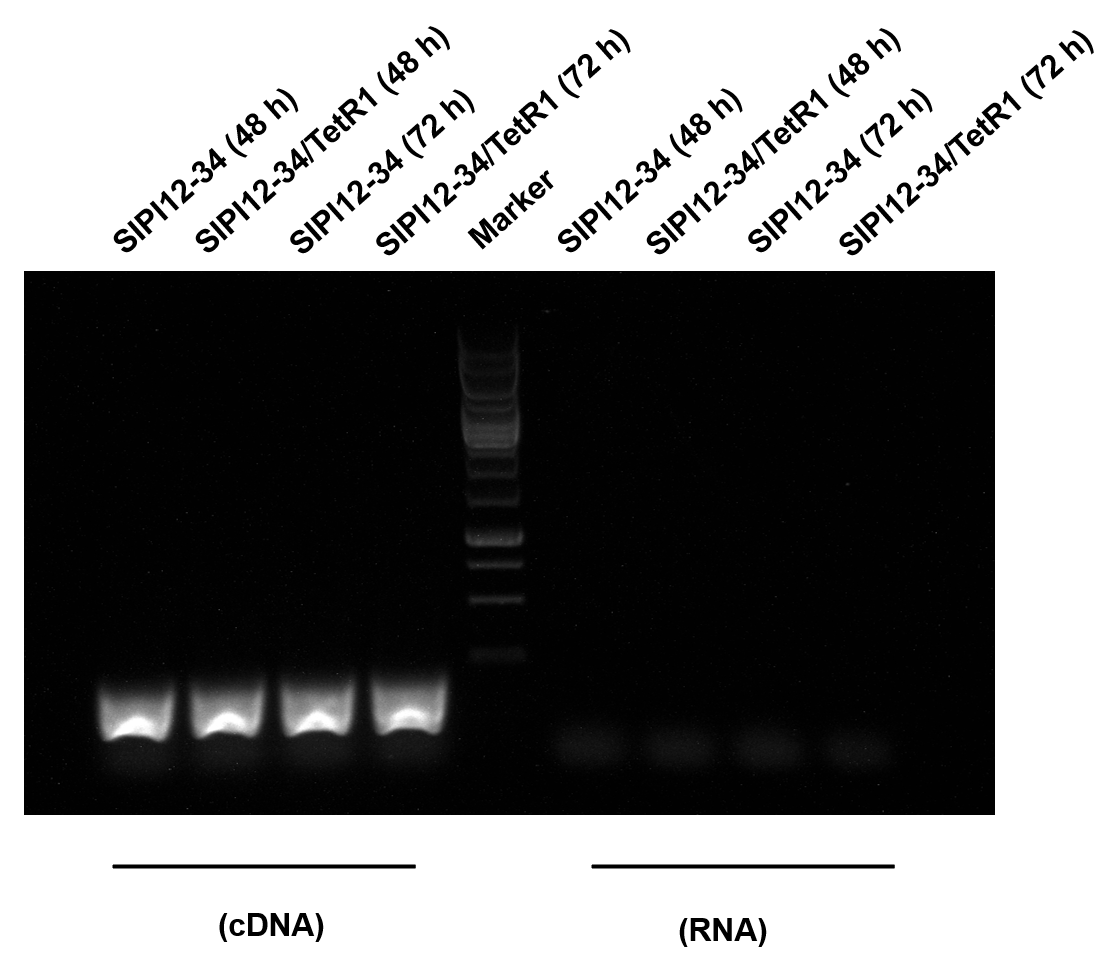


**Fig. S3** The elimination of genomic DNA in obtained RNA of SIPI12-34 and SIPI12-34/TetR1 were confirmed by PCR amplification using the primer pairs 16S1-F/R. In PCR validation, there was no band display using RNA as template, and 200 bp bands were obtained using cDNA as template.


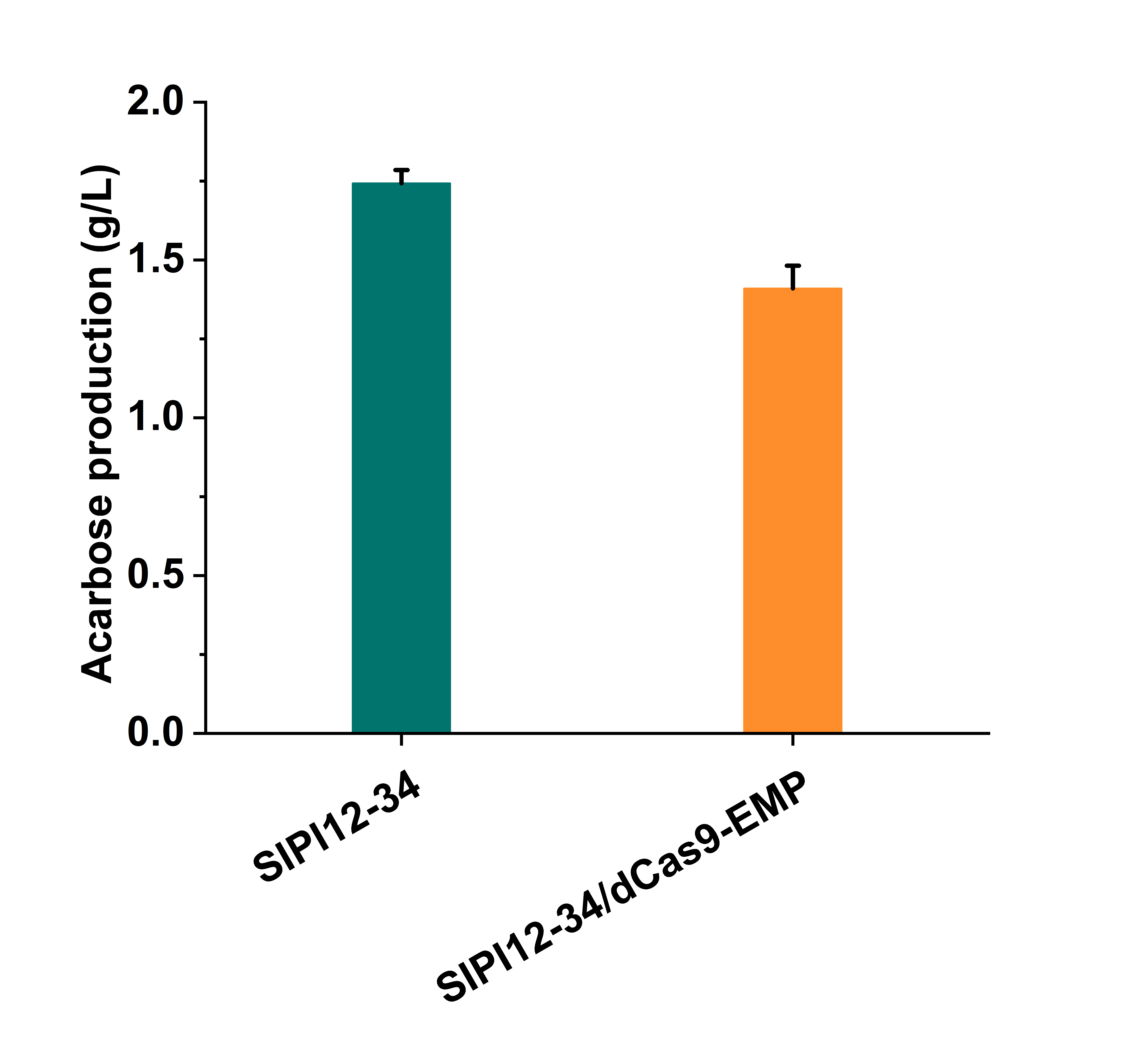


**Fig. S4** Acarbose production profiles of SIPI12-34 and SIPI12-34/dCas9-EMP were analyzed by HPLC. SIPI12-34/dCas9-EMP: Mutant strain in which the expression of key enzyme gene (6-phosphofructokinase) in the glycolytic pathway was inhibited by the CRISPR-dCas9 system.


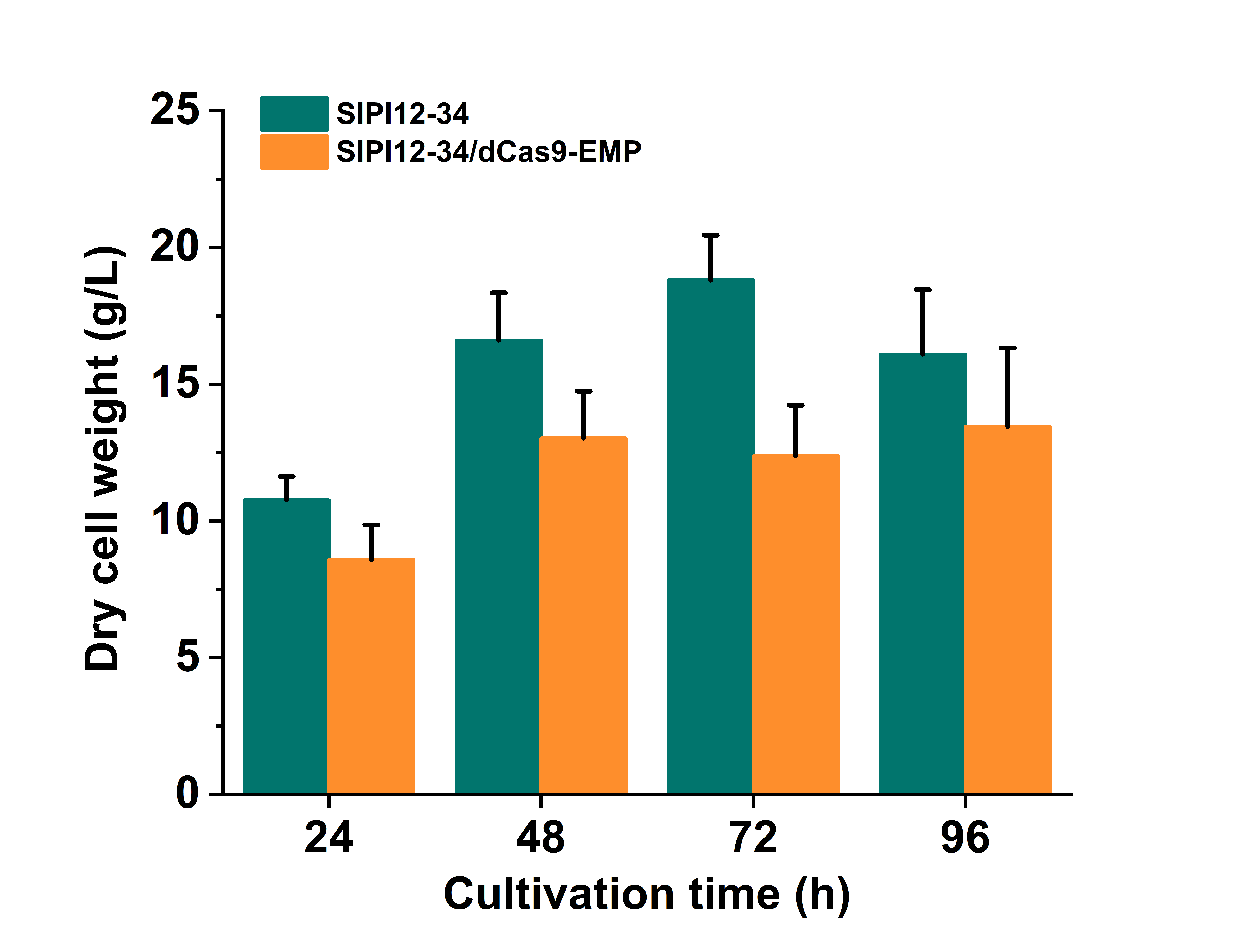


**Fig. S5** Dry cell weights of SIPI12-34 and SIPI12-34/dCas9-EMP at different times during the fermentation stage. Three replicates for each strain at each time point.


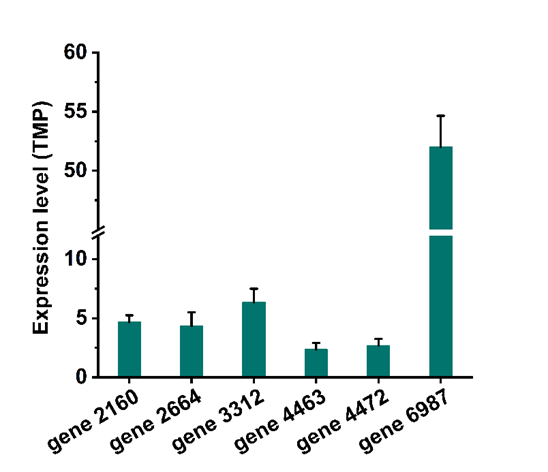


**Fig. S6** The expression of genes encoding glycogen synthase derived from transcriptome sequencing**.** TPM (Transcripts Per Million reads): Number of read sections from a transcript of transcripts per million reading segments.


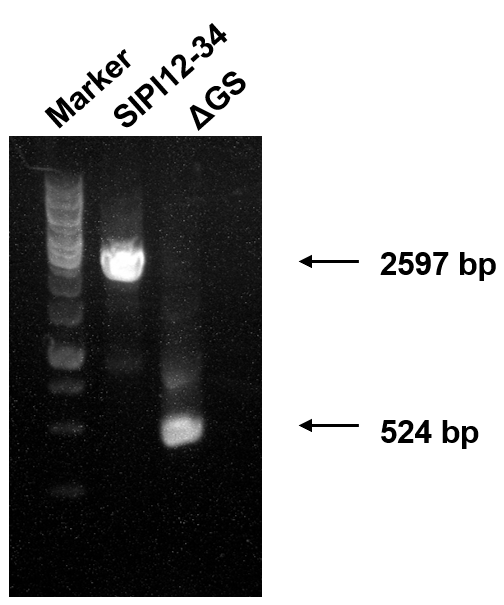


**Fig. S7** PCR validation of ΔGS and SIPI12-34. ΔGS: mutant strain with complete deletion of glycogen synthase gene (2073 bp) in the chromosome of SIPI12-34.


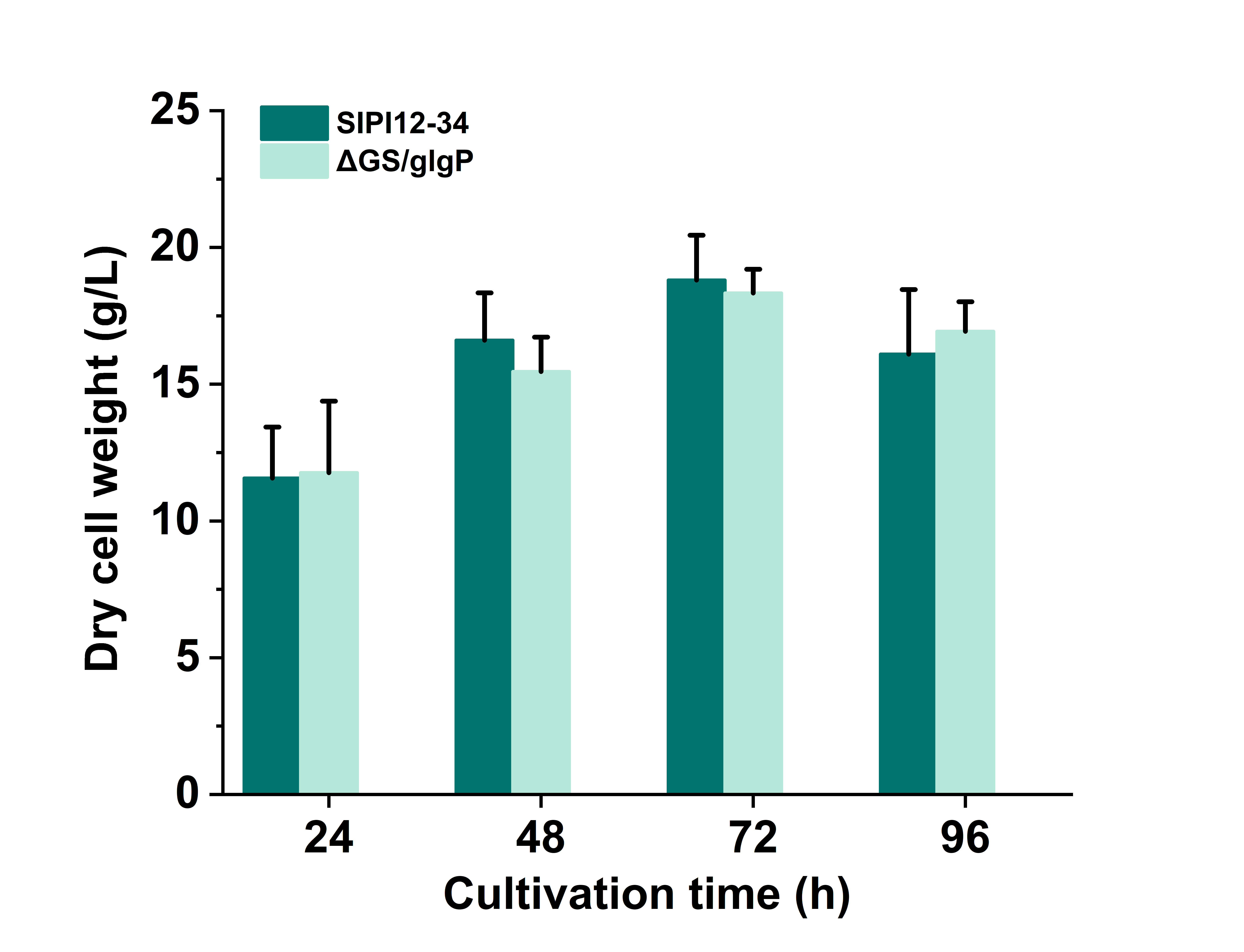


**Fig. S8** Dry cell weights of SIPI12-34 and ΔGS/glgP at different times during the fermentation stage.


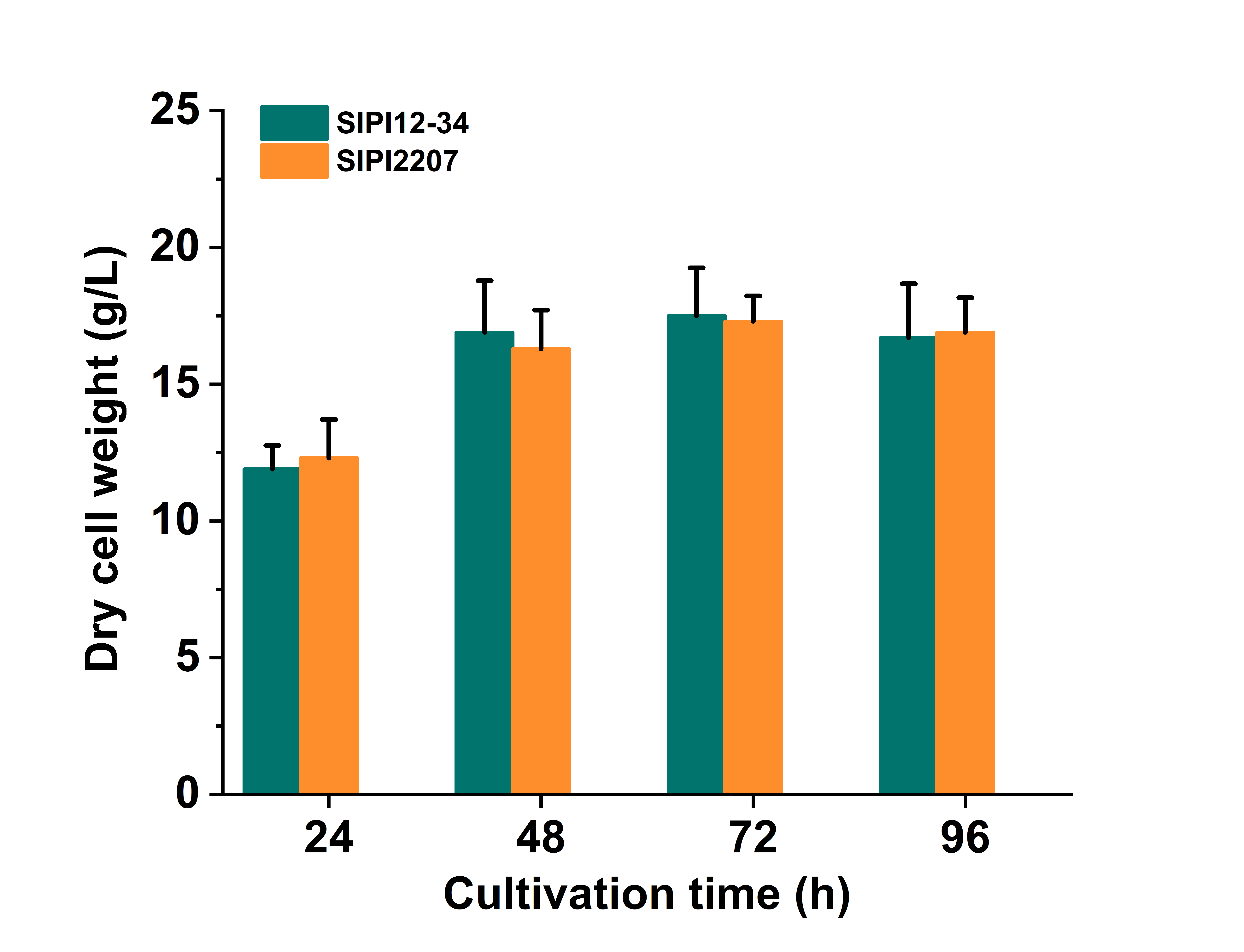


**Fig. S9** Dry cell weights of SIPI12-34 and SIPI2207 at different times during the fermentation stage.


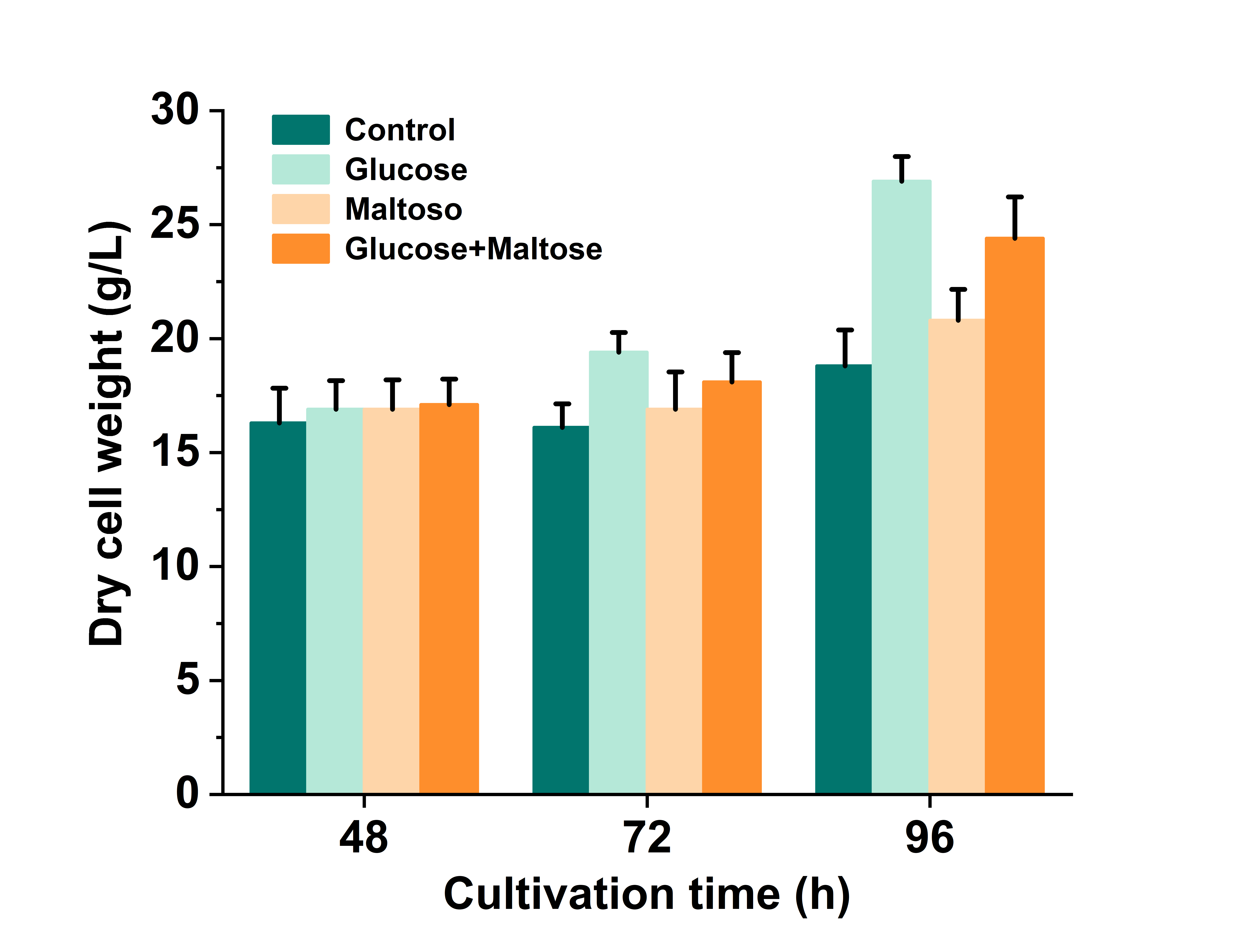


**Fig. S10** Dry cell weights of SIPI2207 with addition of different sugars during the fermentation stage. Control represents no supplementation for SIPI2207. Glucose represents supplementation of 20 g/L glucose for SIPI2207 at 48 h. Maltose represents supplementation of 20 g/L maltose for SIPI2207 at 48 h. Glucose+Maltose represents supplementation of a 10 g/L mixture of glucose and maltose for SIPI2207 at 48 h.


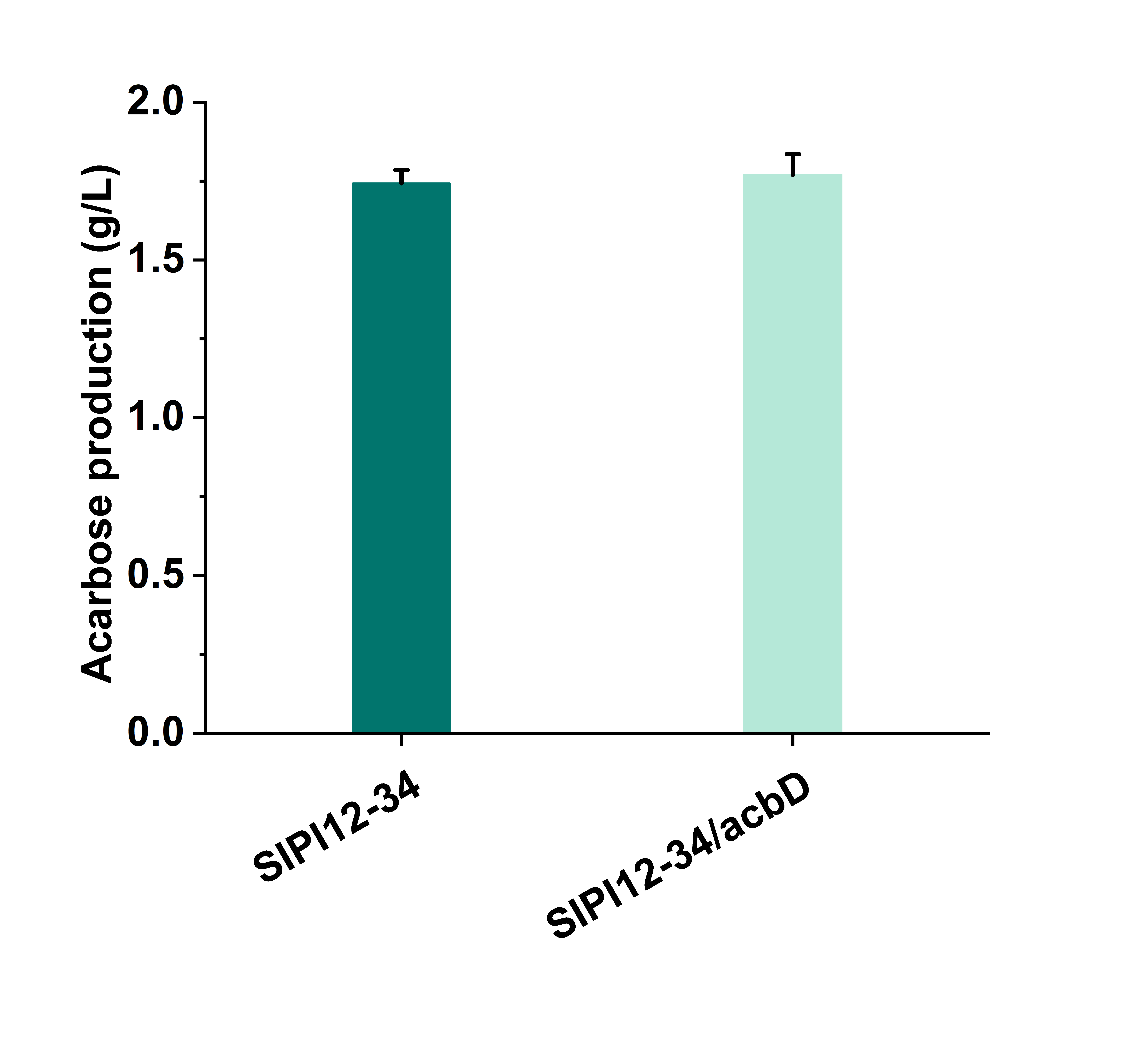


**Fig. S11** Acarbose production profiles of SIPI12-34 and SIPI12-34/acbD were analyzed by HPLC.
